# Lyme Disease Testing Practices, Wisconsin, USA, 2016–2019

**DOI:** 10.3201/eid3107.250009

**Published:** 2025-07

**Authors:** Kiersten J. Kugeler, Erica Scotty, Austin Earley, Alison F. Hinckley, Sarah A. Hook, Courtney C. Nawrocki, Alexandra M. Linz, Jennifer Meece, Anna M. Schotthoefer

**Affiliations:** Centers for Disease Control and Prevention, Fort Collins, Colorado, USA (K.J. Kugeler, A. Earley, A.F. Hinckley, S.A. Hook, C.C. Nawrocki); Marshfield Clinic Research Institute, Marshfield Clinic Health System, Marshfield, Wisconsin, USA (E. Scotty, A.M. Linz, J. Meece, A.M. Schotthoefer)

**Keywords:** Lyme disease, vector-borne infections, Borrelia burgdorferi, bacteria, zoonoses, laboratory testing, surveillance, Wisconsin, United States

## Abstract

Positive laboratory results are increasingly used for Lyme disease surveillance in the United States. We found 6%–15% of persons with a positive test each year tested positive in a prior year; repeat testing frequency increased with patient age. Repeat testing of persons with previous seropositivity could affect surveillance data interpretation.

Lyme disease, a tickborne illness caused by *Borrelia burgdorferi* spirochetes, is the most commonly reported vectorborne disease in the United States (*1*–*3*). Most cases are reported from high-incidence states in the Northeast, mid-Atlantic, and upper Midwest: Connecticut, Delaware, Maine, Maryland, Massachusetts, Minnesota, New Hampshire, New Jersey, New York, Pennsylvania, Rhode Island, West Virginia, Wisconsin, Vermont, and Virginia and the District of Columbia. Laboratory diagnostic tests primarily rely on serology. For decades, the standard approach to serologic testing for Lyme disease has been a standard 2-tier (STTT) reflex algorithm, in which specimens with positive or equivocal results on a first-tier screening assay, usually an enzyme immunoassay, are then tested by immunoblots for IgM and IgG detection to confirm specific antibody reactivity (*4*,*5*). Detectable antibodies typically persist for months to years; therefore, repeat testing is not generally expected to provide clinically relevant information about the resolution of infection or evidence of possible reinfection (*4*,*6*).

Since 2022, a positive laboratory result has been considered sufficient to report Lyme disease cases through public health surveillance in high-incidence states (*7*,*8*). However, relatively little is known about test ordering and repeat testing practices that drive the large volume of positive serologic tests in the United States. That knowledge is integral to accurately interpret Lyme disease surveillance data. We summarized Lyme disease serologic test ordering frequency, positivity rates, patient characteristics, and repeat testing patterns in a health system in Wisconsin, USA, during 2016–2019.

## The Study

The Marshfield Clinic Health System serves north-central Wisconsin, which is a state that has a high incidence of Lyme disease (*3*). For this study, we used laboratory data collated from a larger effort to identify and describe Lyme disease cases in electronic health records in the Marshfield Clinic Health System (*9*).

For analytic purposes, we grouped all Lyme disease tests occurring per person per calendar month into 1 testing episode. We described the frequency of testing episodes and positive results, including repeat testing per person, and calculated 95% Wilson CIs around percentages and χ^2^ tests where appropriate. We used SAS version 9.4 (SAS Institute, https://www.sas.com) for all analyses. The Marshfield Clinic and Centers for Disease Control and Prevention determined that this study was exempt from human subjects research regulations.

During 2016–2019, a total of 42,077 STTT testing episodes occurred among 36,984 unique patients. Test episodes were more common among female (51.5%) than male (48.5%) patients, among persons 50–69 years of age (29.8%), and during May–August (52.6%). We found that 2,911 (6.9%) persons had positive STTT results, and the results varied by age and sex. Positive results were highest among children, male patients, and specimens submitted in the summer months (Table 1; Figure).

**Table 1:** Patient characteristics and positivity rates of Lyme disease serologic testing, USA, 2016–2019*

Characteristic	No. (%) test episodes	% Positive (95% CI)
Patient sex†		
F	21,656 (51.5)	5.8 (5.5–6.1)
M	20,420 (48.5)	8.1 (7.7–8.5)
Patient race/ethnicity		
American Indian/Alaska Native	303 (0.7)	6.9 (4.6–10.4)
Asian	302 (0.7)	8.6 (6.0–12.3)
Black or African American	130 (0.3)	5.4 (2.6–10.7)
Native Hawaiian/other Pacific Islander	28 (0.1)	7.1 (2.0–22.7)
White	37,457 (86.6)	7.1 (6.8–7.3)
>1 race	199 (0.5)	5.5 (3.1–9.6)
Hispanic	623 (1.5)	5.6 (4.1–7.7)
Unknown or not reported	4,035 (9.6)	5.9 (5.2–6.6)
Testing month		
January	1,835 (4.4)	4.6 (3.8–5.7)
February	1,563 (3.7)	4.0 (3.1–5.1)
March	1,746 (4.2)	4.8 (3.9–5.9)
April	2,243 (5.3)	5.1 (4.3–6.1)
May	4,215 (10.0)	5.0 (4.3–5.7)
June	6,229 (14.8)	6.7 (6.1–7.3)
July	6,399 (15.2)	9.6 (8.9–10.4)
August	5,306 (12.6)	9.4 (8.6–10.2)
September	3,696 (8.8)	7.3 (6.5–8.2)
October	3,648 (8.7)	7.2 (6.4–8.1)
November	3,022 (7.2)	5.8 (5.0–6.7)
December	2,175 (5.2)	5.4 (4.5–6.4)

*Results reflect 42,077 test episodes. †One patient missing data on sex.

**Figure:**
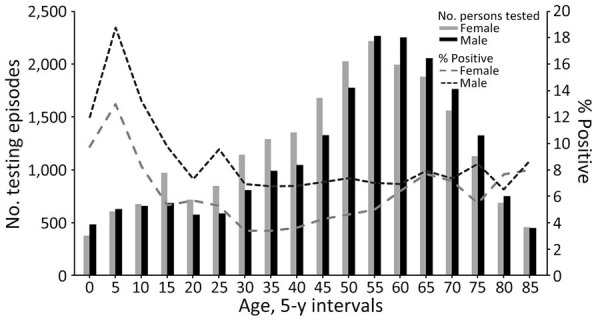
Lyme disease serologic testing episodes and percentage positivity by patient age and sex, Wisconsin, USA, 2016–2019.

Most (88.4%, 32,684) persons had only 1 testing episode during the 4-year period; of those persons, 6.1% had a positive result. Among 34,585 persons with an initial negative serologic test, we noted that 3,892 (11.3%) were tested again during the study period, and 3,711 (95.3%) of those had negative results on all subsequent testing. The frequency of repeat testing after an initial negative test did not differ by patient sex but increased with patient age (p<0.001). Among the 2,084 persons tested again within 12 months of an initial negative serologic testing episode, 110 (5.3%) had subsequent positive STTT results.

The 2,911 positive STTT episodes occurred among 2,580 persons; 450 (17.4%) persons with a positive testing episode were tested again >1 time, at a mean of 12 (median 11, range 1–39) months later. Among those 450 persons, 275 were tested again within 12 months, and 64.4% of that subset remained seropositive. Another 111 persons were tested after 12 months but <24 months after the first positive test, and 55.0% of results from those subsequent testing episodes were positive.

The percentage of positive testing episodes per year that occurred among persons who had tested positive in a prior year ranged from 5.8% in 2017, when only 2016 testing was available, to 15.3% in 2018, when we considered data from 2016 and 2017 (Table 2). The percentage of persons who tested positive in a prior year increased with patient age (p = 0.01); 5.8% of persons with a prior positive result were <15 years of age, 17.4% were 15–44 years of age, 33.3% were 45–64 years of age, and 43.5% were >65 years of age.

**Table 2:** Lyme disease serologic resting episodes and repeat positive results per, Wisconsin, USA, 2016–2019*

Year	No. serologic testing episodes (% positive)	No. positive in a prior year/no. positive (%)	No. (%) persons		
			Prior positive in 2016	Prior positive in 2017	Prior positive in 2018
2016	11,878 (6.7)	NA	NA	NA	NA
2017	12,150 (7.9)	54/928 (5.8)	54 (5.8)	NA	NA
2018	9,444 (6.2)	86/563 (15.3)	33 (5.9)	56 (10.0)	NA
2019	8,605 (6.6)	81/545 (14.9)	26 (4.8)	33 (6.1)	37 (6.8)

*Some tested positive in >1 prior year. Thus, year-specific columns sum to more than the total number of persons in that year who had previously tested positive.

Patterns of positive laboratory tests mirrored the demographics and seasonality of Lyme disease as demonstrated through decades of public health surveillance, having more positive results among male persons, among children, and during the summer months (*2*,*3*). However, testing more commonly occurred among older adults and female persons, and the largest discrepancy we observed between test ordering frequency and positive results occurred among middle-aged women, as documented elsewhere (*10*,*11*). Excess negative tests among this group might indicate greater frequency of healthcare visits for signs and symptoms that overlap with or have the potential to be misdiagnosed as Lyme disease or possible differences in the serologic response to *B. burgdorferi* by age and sex (*12*,*13*). Additional studies are needed to understand the drivers of increased testing frequency and lower positivity rate in this group.

In 2022, the Council of State and Territorial Epidemiologists implemented a revised Lyme disease surveillance case definition in high-incidence states on the basis of positive laboratory results alone. The revision in the subset of the most highly affected states was intended to reduce human resource burden on health departments in high-incidence states and improve standardization of data captured across states (*14*). However, by eliminating the requirement for a concurrent clinically relevant illness, persons might now be captured as Lyme disease cases when their positive test does not reflect incident Lyme disease. The first year of data ascertained under the new case definition showed that reliance on only positive laboratory results in high-incidence states resulted in a disproportionate increase in reported cases among persons >65 years of age (*8*).

The first modified 2-tier testing assays were cleared by the US Food and Drug Administration in 2019, and commercial uptake gradually occurred thereafter (*15*). The patterns associated with standard 2-tier testing positivity we report might not be generalizable to characteristics associated with testing on modified 2-tier assays, all of which have different performance characteristics from each other and standard 2-tier assays.

## Conclusions

Although serologic testing on persons who previously tested positive for Lyme disease is often not clinically relevant because antibodies to *B. burgdorferi* may persist years after infection, we found up to 15% of persons with positive serologic tests per year also tested positive in a previous calendar year. The frequency of such repeat positives increased with patient age. 

Our findings provide evidence that a percentage of reported Lyme disease cases each year may not reflect incident Lyme disease and that percentage increases with patient age and thus might explain the substantial increase in reported Lyme disease cases among older adults beginning in 2022. Knowledge of the frequency and characteristics of repeat testing among persons who have previously tested positive improves our ability to interpret national Lyme disease surveillance data in a more appropriate context.
